# Intrinsisch lichtempfindliche Ganglienzellen Die physiologische nicht-visuelle Wirkung von Licht

**Published:** 2021-09

**Authors:** Manuel Spitschan

**Affiliations:** Oxford

## Abstract

Zusätzlich zum Sehen beeinflusst Licht grundlegend unsere Physiologie und unser Verhalten durch die nicht-visuellen Nervenbahnen im Gehirn, die unsere innere Uhr kontrollieren. Diesen Wirkungen liegen maßgeblich die intrisisch lichtempfindlichen Ganglienzellen zu Grunde, die Photopigment Melanopsin enthalten, das primär auf kurzwelliges Licht reagiert. Die nicht-visuellen Lichtwirkungen und die damit verbundenen sensorischen und zentralen Mechanismen bleiben ein aktives und noch offenes Forschungsthema.

## Summary

In addition to enabling us to see, light fundamentally impacts on our physiology and behaviour through the non-visual pathways in the brain that control our circadian clock. These effects are largely mediated by the intrisically photosensitive retinal ganglion cells (ipRGCs) which express the short-wavelength-sensitive photopigment melanopsin. The non-visual effects of light and the underlying sensory and central mechanisms are an active and open area of investigation.

## Intrinsisch lichtempfindliche Ganglienzellen

Nur durch Licht, das auf die Netzhaut fällt, ist die visuelle Wahrnehmung der Welt um uns möglich. Die Zapfen und Stäbchen der Netzhaut verarbeiten Lichtreize, die im visuellen System zur Farb-, Raum- und Bewegungswahrnehmung führen. Bis Ende der 1990er Jahre bestand die Annahme, dass die Zapfen und Stäbchen die einzigen lichtempfindlichen Zellen in der Netzhaut sind. Die Entdeckung der sogenannten intrinsisch lichtempfindlichen Ganglienzellen („intrinsically photosensitive retinal ganglion cells“, ipRGC) stellte diese Annahme auf den Kopf: ipRGC stellen einen weiteren lichtempfindlichen Mechanismus im Auge dar, der unabhängig von den Zapfen und Stäbchen abläuft [7, 8, 11, 14, 17].

Intrinsisch lichtempfindliche Ganglienzellen machen weniger als 5 % der retinalen Ganglienzellen aus. Sie sind über die ganze Netzhaut verteilt. Im Unterschied zu den anderen Ganglienzellen enthalten ipRGC das lichtempfindliche Protein Melanopsin. Ihre Funktion ist nicht die Bild- oder Mustererkennung oder das Farbensehen, sondern die Wahrnehmung der Umgebungshelligkeit.

## Empfindlichkeitsmaximum von Melanopsin liegt bei 490 nm

Die ipRGC reagieren insbesondere auf kurzwelliges Licht. Die spektrale Empfindlichkeit von Melanopsin liegt bei 480 nm, also im Blaubereich. Da die Linse und die okulären Medien das Licht allerdings auch noch einmal filtern, bevor es auf die Netzhaut fällt, wird als effektives Empfindlichkeitsmaximum ein Wert von 490 nm angenommen. Dies gilt modellhaft für einen 32 Jahre alten Menschen. Das Maximum verschiebt sich mit dem Alter hin zu längeren Wellenlängen [21].

## Nicht bildformendes Sehen wird über ipRGC gesteuert

Prinzipiell bewerkstelligt das menschliche Auge zwei sehr unterschiedliche Aufgaben: Einerseits nehmen wir über das sogenannte „bildformende Sehen“ unsere Umwelt wahr. Daneben laufen aber auch Prozesse des „nicht bildformenden Sehens“ ab, derer wir uns nicht bewusst werden. Eine der Hauptaufgaben ist es dabei, die Körperfunktionen an die Lichtverhältnisse der Umgebung anzugleichen [11a].

Hier kommen die intrinsisch lich-tempfindlichen Ganglienzellen ins Spiel. Sie beeinflussen weitgehend die physiologische Antwort auf Licht. Gemeint ist damit, dass angenommen wird, dass die ipRGC keine Informationen zu unserer visuellen Wahrnehmung beisteuern – oder neuroanatomisch betrachtet – nicht über die kortikale Sehbahn kommunizieren. Stattdessen beeinflusst die Aktivität der ipRGC maßgeblich wichtige physiologische Kontroll-mechanismen.

## Innere Uhr und zirkadianer Rhythmus

**Abbildung 1:**
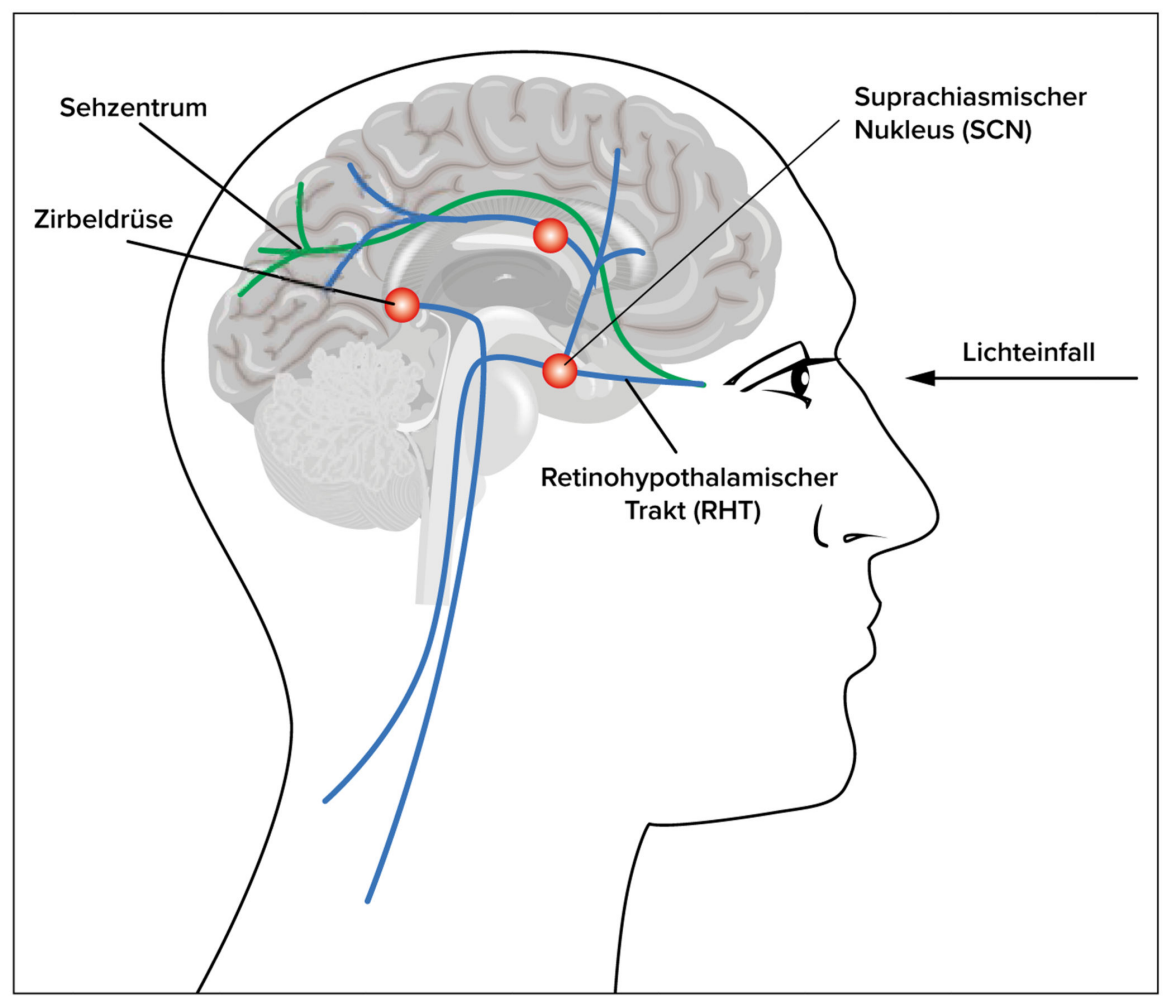
Stäbchen und Zapfen übertragen den optischen Reiz via Sehnerv ins Sehzentrum des Gehirns (grüner Pfad). Die ipRGC leiten die Signale der Netzhaut über den retinohypothalamischen Trakt („retinohypothalamic tract“, RHT) an den Nucleus suprachiasmaticus („suprachiasmatic nucleus“, SCN) im Zwischenhirn weiter (blauer Pfad).

Stäbchen und Zapfen übertragen den optischen Reiz via Sehnerv ins Sehzentrum des Gehirns (Netzhaut > Corpus genivulatum laterale (LGN) > Sehrinde). Die ipRGC leiten die Signale der Netzhaut über den retinohypothalamischen Trakt („retinohypothalamic tract“, RHT) an den Nucleus suprachiasmaticus („suprachiasmatic nucleus“, SCN) im Zwischenhirn weiter (Abbildung 1). Der SCN ist das nervliche Substrat unserer inneren, biologischen Uhr. Nervenzellen im SCN kodieren die Lichtintensität unserer Umgebung und oszillieren auch in Abwesenheit von externen Reizen innerhalb einer Periode, die fast 24 Stunden beträgt (zirkadian) [10]. Der SCN kann als Dirigent eines Orchesters verstanden werden, der den Takt für den restlichen Körper gibt. Inzwischen ist bekannt, dass jede Zelle eine „molekulare Uhr“ enthält.

Die Verschaltung von Auge und unserer inneren Uhr macht Licht für unsere Leben so wichtig: Unsere innere Uhr wird durch Lichtexposition mit der Erdumdrehung synchronisiert und hat so einen 24-Stunden-Rhythmus. Andererseits kann Lichtexposition zur falschen Zeit unsere innere biologische Uhr durcheinanderbringen: Licht am Abend und in der Nacht kann die Synchronisierung stören und zu falschen „Tag“- Signalen führen.

### Produktion von Melatonin ist lichtabhängig

Akut wirkt sich Lichtexposition am Abend auf unseren Hormonhaushalt und speziell auf das „Schlafhormon“ Melatonin aus, das am Tag nicht produziert wird und dessen Produktion zirka 1–3 Stunden vor der habituellen Schlafenszeit steil ansteigt. Schon Raumlicht kann die Produktion von Melatonin hemmen.

Vor kurzem wurde außerdem belegt, dass es sehr große interindividuelle Unterschiede in der Empfindlichkeit auf Abendlicht gibt: So lagen die empfindlichsten und unempfindlichsten Personen um den Faktor 60 auseinander [16]. Es ist noch unklar, ob sich diese Unter-schiede in der physiologischen Licht-wirkung auf genetisch bedingte Unter-schiede im Melanopsinpigment zurück-führen lassen.

### Mit Licht kann man die innere Uhr umstellen

In Laborstudien wurde außerdem nachgewiesen, dass mit Lichtreizen die innere Uhr dauerhaft verschoben werden kann [6, 9, 26]. Je nachdem, wann die Lichtexposition stattfindet, kann die Uhr nach hinten oder vorne gestellt werden. Licht am Morgen verschiebt den Zeiger der Uhr nach vorne [12, 13, 15]. Licht am Abend hingegen verschiebt den Zeiger nach hinten. Dabei gilt generell: Je heller das Licht, desto größer die physiologische Wirkung.

Studien mit monochromatischem Licht, also Licht aus nur einem kleinen Wellenlängenbereich, konnten nachweisen, dass sowohl die Melatoninproduktion als auch die Wirkung auf die innere Uhr maßgeblich von Melanopsin und den ipRGC gesteuert werden. Eine vor kurzem veröffentlichte Übersichts-arbeit hat die dazu existierende Literatur zusammengefasst und dargelegt, dass dieser Ansatz die physiologische Antwort auf langanhaltende Lichtreize ausreichend beschreibt [2].

## Licht ist mehr als Beleuchtung unserer Umgebung

Wir setzen uns im Alltag Licht ganz unterschiedlichen Ursprungs aus. Die Hauptlichtquelle stellt dabei Tageslicht dar, also Licht, das von der Sonne ausgeht und atmosphärisch gefiltert und verteilt wird. Im Büro und im Heim gibt es unterschiedliche Lichtquellen. Speziell die Beleuchtung mit LED ist mittlerweile weit verbreitet.

**Abbildung 2:**
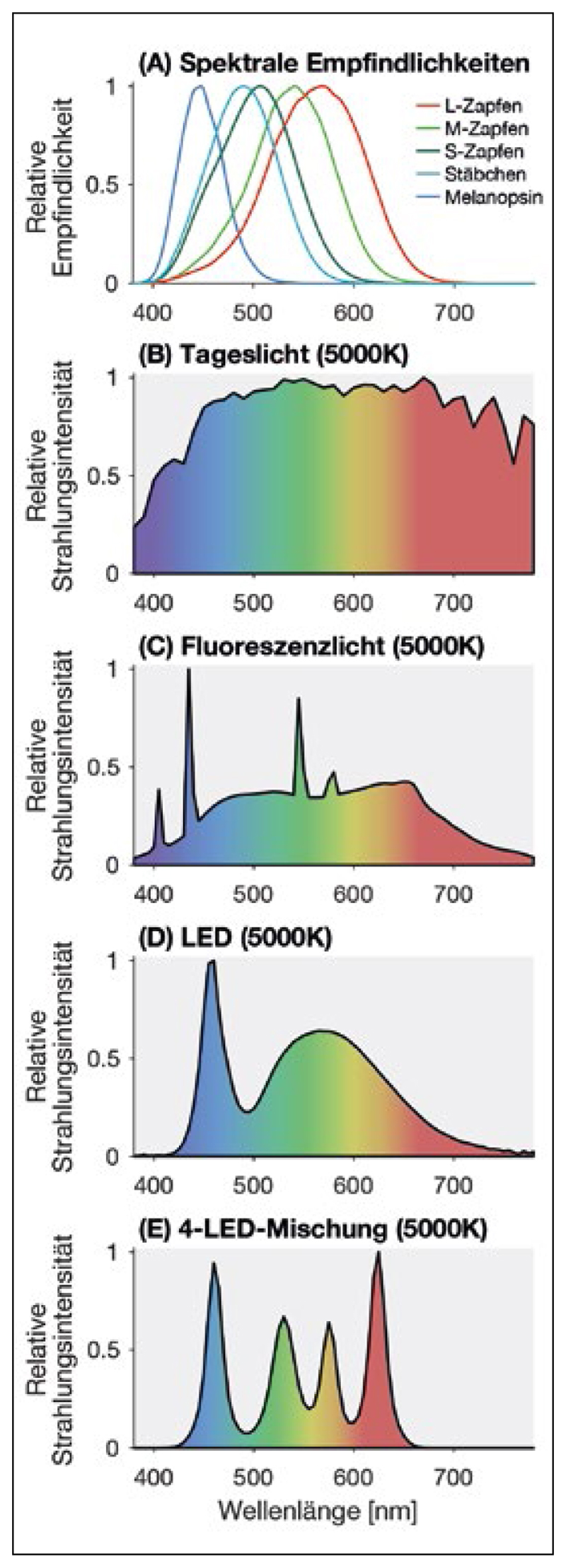
In Abbildung 2 B–E sind vier Spektren dargestellt, die alle gleich aussehen, d. h. die Zapfen, die für das Farbensehen zuständig sind, werden gleichermaßen angesprochen durch die unterschiedlichen Spektralverteilungen, Tageslicht hat ein breites Spektrum (B), während sich künstliches Licht im Spektrum je nach Lichtquelle stark unterscheidet.

Natürliches und künstliches Licht unterscheiden sich insbesondere in dem ihm zugrunde liegenden Spektrum. In Abbildung 2 sind vier Spektren darge-stellt (B–E), die alle gleich aussehen, d. h. die Zapfen, die für das Farbensehen zuständig sind, werden gleichermaßen angesprochen durch die unterschiedlichen Spektralverteilungen, Tageslicht hat ein breites Spektrum (Abbildung 2B), während sich künstliches Licht im Spektrum je nach Lichtquelle stark unterscheidet.

Abgesehen von der Intensität machen die ipRGC und allgemeinen die Photozeptoren allerdings keinen Unterscheid zwischen den einzelnen Spektren. Das liegt daran, dass ein einzelnes lichtempfindliches Molekül nicht zwischen unterschiedlichen Wellenlängen unterscheiden kann: Das von Rushton entwickelte Univarianz-Prinzip besagt, dass die die Erregung einer Sehzelle nur von der Absorption eines Photons abhängt [18]. Die Absorption hingegen folgt der spektralen Empfindlichkeit. So haben unterschiedliche Opsine – wie z. B. Melanopsin – ein Empfindlichkeits-maximum, aber nach der Absorption des Photons kann nicht mehr festge-stellt werden, welche Wellenlänge dieses Photons hatte. Dies bedeutet, dass für die Photorezeptoren Licht unter-schiedlicher Spektren die exakt gleiche Wirkung haben kann.

### Photic history

Die Lichtexposition löst komplexe Vorgänge aus, die über die bloße Wirkung auf die fünf Photoerzeptor-Typen hinausgehen. Unter dem Stichwort „photic history“ werden in der Chrono-biologie solche Lichtwirkungen erfasst, die in der Vergangenheit stattgefunden haben und immer noch nicht-visuell nachwirken. Ganz konkret gehört dazu die Tatsache, dass Lichtexposition am Tag uns gegenüber Licht am Abend in Hinblick auf die melatoninsupprimierende Wirkung von Licht unempfindlicher machen kann.

Wo genau neuronal diese Anpassung an die „Lichthistorie“ stattfindet, ist noch nicht bekannt. Für die zeitlich abhängige Lichtexposition, die physiologisch auch mit den Augenbewegungen in Zusammenhang steht, wurde der Terminus „spektrale Diät“ eingeführt – nicht zuletzt, um auch auf die Veränderbarkeit der alltäglichen Lichtexposition hinzu-weisen [25].

### Die ipRGCs als Integratoren

Obwohl die ipRGC Licht ganz unab-hängig von den Zapfen und Stäbchen verarbeiten können, ist nachgewiesen, dass synaptische Verbindungen zwischen den Photorezeptoren bestehen, die die Aktivität der ipRGC beeinflussen können. So werden die ipRGC zu „Integratoren“, in dem sie die Signale aller Photorezeptoren im Auge bündeln und so eine sehr große Spanne von Lichtintensitäten abbilden können.

### Einfluss der Zapfen auf die ipRGC

Vor einigen Jahren konnte eine Studie belegen, dass S-Zapfen, wenn diese mit Flimmerlicht selektiv angesprochen werden, die Pupillengröße, die auch von den ipRGC kontrolliert wird, negativ beeinflusst [22]. Das heißt, Lichtreize, die die S-Zapfen aktivieren, führen zu einer Weitung der Pupille, also einer paradoxen Pupillenreaktion [19]. In einer Folge-studie konnte allerdings keine offen-sichtliche Wirkung der S-Zapfen auf die Melatoninproduktion nachweisen [20].

### Einfluss der Stäbchen auf die ipRGC

Auch der Einfluss der Stäbchen, die viel lichtempfindlicher als die Zapfen sind, auf die ipRGC, ist noch nicht genau geklärt. In einer Pilotstudie zeigten Achromaten trotz sehr hoher Blendemp-findlichkeit und der durch Lichtschutz-brillen bedingten Herunterstufung der Lichtexposition einen normalen Schlaf-Wach-Rhythmus und eine auch weitgehend normale Melatoninproduktion [23]. Weitere Studien, die die Rolle der Stäbchen erforschen, sind in Planung.

## Empfehlungen für den Alltag

Eine internationale Expertengruppe hat vor kurzem Empfehlungen entwickelt, welche Beleuchtungsstärken für welche Tageszeiten und Tätigkeiten sinnvoll sind [1].

Die Empfehlungen schlagen vor:

▪ >250 lux („melanopic equivalent daylight illuminance“, melanopic EDI) in Augenhöhe am Tag (idealerweise durch Tageslicht),
▪ <10 lux melanopic EDI am Abend und
▪ <1 lux melanopic EDI in der Nacht (idealerweise allerdings komplett dunkel)

Diese Empfehlungen stellen Richtwerte dar: Die Umsetzung und Umsetzbarkeit muss im Zweifelsfalle messtechnisch geprüft werden. Für den Alltag gilt das Mantra: *Helle Tage und dunkle Nächte*.

## Box

**Empfindlichkeitskurven der fünf Photorezeptor-Typen**

2018 hat die Internationale Beleuchtungskommission (CIE; Commission Internationale de l’Éclairage), die unter anderem für die Entwicklung nationaler und internationaler Normen zuständig ist, die Empfindlichkeitskurven der fünf Photorezeptor-Typen im menschlichen Auge in der Norm CIE S 026/E:2018 standardisiert [3]. Dies vereinfacht die messtechnische Bestimmung der physio-logischen Wirkung von Licht, in dem Lichtreize mit netzhautbezogenen und physiologisch relevanten Größen beschrieben werden können. Die Berechnungen können in einer von der CIE veröffentlichten Excel-Toolbox [4, 5] und auf der neuen Plattform https://luox.app/ durchgeführt werden [24].

CIE S 026/E:2018 stellt auch eine weitere Größe vor: Die „melanopic equivalent daylight illuminance“ eines Spektrums, kurz melanopic EDI oder auf deutsch die „melanopische tageslichtäquivalenten Beleuchtungsstärke“. Melanopic EDI gibt an, welche photopische Beleuchtungsstärke (in lux) ein Tageslichtspektrum (D65, 6500K) sein muss, um die gleiche melanopische Wirkung wie das Spektrum zu haben.
